# In Vitro Assessment of Electrospun PVP+AgNPs Scaffolds for Bioactive Medical Use

**DOI:** 10.3390/ijms26189114

**Published:** 2025-09-18

**Authors:** Ileana Ielo, Luana Vittoria Bauso, Antonio Laezza, Paola Campione, Luigi Fabiano, Martina Pastorello, Andreana Marino, Alessandro Laurita, Antonietta Pepe, Brigida Bochicchio, Giovanna De Luca, Grazia Maria Lucia Messina, Giovanna Calabrese

**Affiliations:** 1 Department of Chemical, Biological, Pharmaceutical and Environmental Sciences (ChiBioFarAm), University of Messina, Viale F. Stagno d’Alcontres 31, 98166 Messina, Italy; ileana.ielo1@unime.it (I.I.); luanavittoria.bauso@unime.it (L.V.B.); martina.pastorello@studenti.unime.it (M.P.); andreana.marino@unime.it (A.M.); 2 Department of Basic and Applied Sciences (DISBA), University of Basilicata, Via Ateneo Lucano 10, 85100 Potenza, Italy; antonio.laezza@unibas.it (A.L.); alessandro.laurita@unibas.it (A.L.); antonietta.pepe@unibas.it (A.P.); brigida.bochicchio@unibas.it (B.B.); 3 Department of Chemical Sciences, University of Catania and CSGI, Viale A. Doria 6, 95125 Catania, Italy; paola.campione@phd.unict.it (P.C.); luigi.fabiano@phd.unict.it (L.F.); gml.messina@unict.it (G.M.L.M.)

**Keywords:** bioactive scaffolds, silver nanoparticles, electrospun scaffolds, biocompatibility, antibiofilm activity

## Abstract

Chronic wounds and post-operative complications generate significant biomedical challenges due to impaired tissue regeneration and persistent microbial infections, often aggravated by biofilm formation and antibiotic resistance. To address these issues, this study investigates the development and in vitro evaluation of electrospun polyvinylpyrrolidone (PVP) scaffolds embedded with silver nanoparticles (AgNPs), designed as multifunctional bioactive platforms for wound healing and implant applications. AgNPs were synthesized and uniformly incorporated into the PVP matrix using optimized electrospinning parameters, harnessing their antimicrobial and anti-inflammatory properties alongside the hydrophilicity, biocompatibility, and chemical stability of PVP. Structural and mechanical characterization, including Transmission Electron Microscopy (TEM) and Atomic Force Microscopy (AFM), homogenous nanoparticle dispersion, and favorable mechanical properties, such as Young’s modulus. In vitro cytotoxicity assays with fibroblast cell lines demonstrated good biocompatibility, while antibiofilm activity against *Staphylococcus aureus* revealed significant microbial inhibition. Overall, electrospun PVP+AgNPs scaffolds demonstrate strong potential as multifunctional biomaterials for wound healing and implant coating due to their synergistic capacity to support tissue regeneration and inhibit infection. These promising results highlight the need for further in vitro and in vivo investigation to confirm their therapeutic efficacy, biocompatibility, and long-term stability in physiological environments.

## 1. Introduction

Effective tissue regeneration remains a central challenge in biomedical science, particularly in the treatment of chronic wounds and post-operative complications [1]. The human body’s natural repair mechanisms, including cell proliferation, migration, angiogenesis, and extracellular matrix (ECM) remodeling, are often compromised in pathophysiological conditions such as diabetic ulcers, ischemic injuries, and chronic infections [2]. These impairments contribute to prolonged inflammation and impaired re-epithelialization, which in turn promote opportunistic microbial colonization, often resulting in biofilm formation [3]. Biofilms are communities of microorganisms in which microbial cells, attached to a biotic or abiotic surface, are enclosed in a matrix of self-produced exopolymeric substances difficult to eradicate [4]. These biofilms create a physical and immunological barrier that perpetuates local immune dysregulation and hinders tissue regeneration, ultimately contributing to wound chronicity and increasing the risk of systemic infection [5]. As a result, patients experience extended recovery periods, an increased risk of morbidity, and substantial financial and resource burdens on global healthcare systems [6]. Traditional therapeutic strategies, while beneficial, have demonstrated a limited effectiveness in addressing the complex interplay between microbial invasion and impaired tissue regeneration [7]. Further aggravating the situation is the rise in antibiotic-resistant bacterial strains, which diminishes the efficacy of conventional treatments [8]. Poor integration of biomaterials with host tissue also restricts their regenerative capacity, leaving clinicians in search of advanced solutions. To overcome these limitations, the focus has increasingly shifted toward the development of innovative biomaterials engineered to interact with biological environments in a multifunctional and dynamic manner [9,10]. Accordingly, the development of advanced biomaterials capable of promoting tissue regeneration while providing effective antimicrobial protection has become a critical research focus. Nanotechnology, in particular, has developed innovative strategies in scaffold design by allowing the precise control over material architecture and bioactivity at the molecular level [11,12,13]. Nanostructured scaffolds not only mimic the native ECM but also offer the ability to deliver therapeutic agents, including antimicrobial nanoparticles. These smart platforms hold promise for accelerating wound closure, enhancing cellular infiltration, and mitigating infection risk, making them pivotal in the future of regenerative medicine [14]. In this context, metallic nanoparticles have drawn considerable interest due to their unique physicochemical properties that enable a wide array of applications from materials chemistry to nanomedicine [15]. In this framework, silver nanoparticles (AgNPs) stand out for their broad-spectrum antimicrobial activity, anti-inflammatory effects, biocompatibility, and ability to modulate cellular behavior [16]. These properties make AgNPs highly versatile for a wide spectrum of biomedical applications, including medical dressings, biosensors, anticancer therapies, implant coatings, and controlled drug delivery systems [17,18]. To address the dual challenge of infection and impaired healing, incorporating AgNPs within bioactive scaffolds has been explored as a promising strategy. The nanoparticles exhibit an enhanced interaction with microbial membranes due to their small size and surface reactivity, while sustained silver ion release ensures prolonged antibacterial performance [19,20]. When embedded into nanofibrous scaffolds, AgNPs emulate the ECM’s architecture, promoting cell adhesion, nutrient exchange, and tissue regeneration [21].

Polyvinylpyrrolidone (PVP), a hydrophilic and biocompatible polymer, offers ideal characteristics for electrospun scaffolds, including chemical inertness, thermal stability, water solubility, non-toxic behavior, and pH resistance [22]. Its mechanical strength and processability make it especially suitable for nanoparticle incorporation via electrospinning, a method that produces high-surface-area fibers, favoring cellular interactions and wound healing. Studies have demonstrated that antimicrobial and antibiofilm enhancement can be obtained in electrospun materials through two primary strategies: post-electrospinning surface coating of the scaffolds, and direct incorporation of AgNPs into the polymer solution before electrospinning [23,24,25,26]. While the latter approach ensures a more homogeneous nanoparticle distribution, it is often limited by AgNPs aggregation, which diminishes the antibacterial efficacy [27,28]. This challenge is mitigated by employing polymers like PVP as capping agents to stabilize nanoparticles and preserve their functional performance [29]. In fact, this polymer can bind the nanoparticles surface through various modes, e.g., by exploiting the known ability of coordinating atoms such as oxygen and nitrogen, as well as sulfur and phosphorus, to bind the various noble metals [30,31,32], favoring the passivation of the nanoparticles surface, and modulating the properties of the resulting nanomaterial.

In this study, we aim to fabricate and evaluate electrospun PVP scaffolds embedded with AgNPs for use as a bioactive component in wound dressings and medical applications. We were able to assess scaffold morphology, nanoparticle dispersion, in vitro cytocompatibility with fibroblast cells, and antibiofilm activity against *Staphylococcus aureus (S. aureus)* and *Klebsiella pneumoniae* (*K. pneumoniae*) with the broader goal of advancing multifunctional materials for wound care and implant applications.

## 2. Results and Discussion

### 2.1. AgNPs Synthesis and Characterization

**Figure 1:**
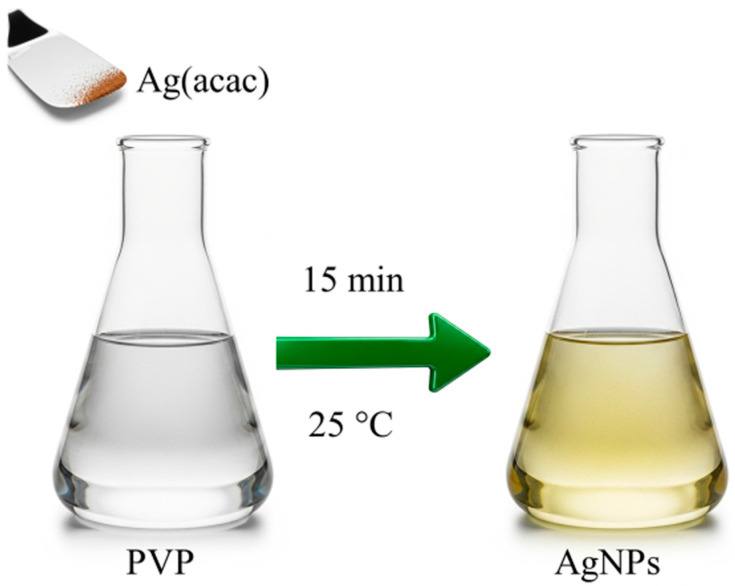
Schematic representation of PVP-functionalized silver nanoparticles (AgNPs) synthesis.

The synthesis of AgNPs was performed using a straightforward and environmentally friendly method, visually summarized in Figure 1. This method leverages the spontaneous thermal decomposition of silver(I) acetylacetonate (Ag(acac)) in water and at room temperature, simplifying the synthesis process and lowering its environmental impact by eliminating harsh chemical reductants [33]. Since the AgNPs will be embedded within PVP electrospun fibers, this polymer is also used as a capping agent. This step will not only enhance the stability of the nanoparticles but also significantly improve their compatibility with the PVP solution used for electrospinning.

AgNPs were synthesized in the presence of the polymer by simply dissolving Ag(acac) into a 0.5 wt% PVP solution in water, as indicated by the starting colorless solution turning pale yellow in a few minutes. The effectiveness of this capping strategy is evident in the absence of aggregation during purification and the improved processability of the AgNPs. Unlike uncapped nanoparticles, the PVP-capped ones can be dried and then easily redissolved in both water and ethanol.

**Figure 2:**
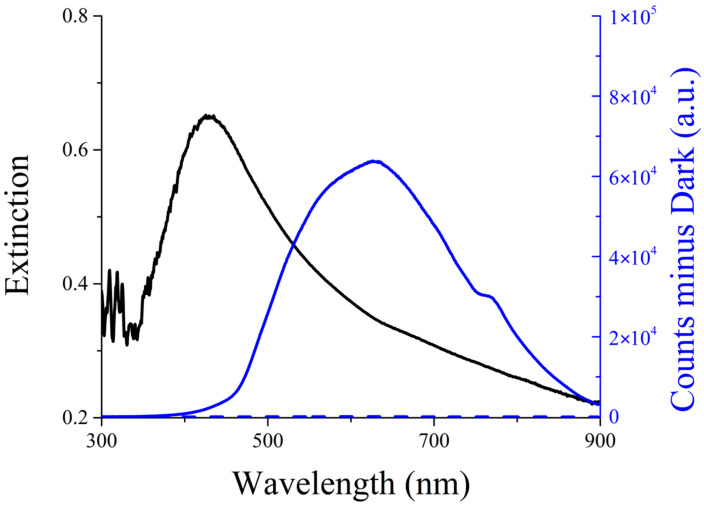
UV-Vis extinction (black line) and Resonance Light Scattering (RLS; solid blue line) spectra of AgNP solutions in water. The RLS from UPW is shown for comparison as a dashed blue line. Experimental conditions: Ag concentration 0.02 mg/mL.

UV-Vis extinction (absorption + scattering) spectroscopy confirms the successful formation of silver nanoparticles, also in the presence of PVP. As shown in Figure 2 (black line), the AgNPs sample exhibits a broad plasmonic band centered at approximately 420 nm, a characteristic typically observed in noble metal nanoparticles. The extinction band presents a tail at its red edge due to the presence of scattered light, as confirmed by the corresponding peak in the Resonance Light Scattering spectrum (Figure 2, solid blue line) [34,35].

The long-term stability of the AgNPs nanocomposite in an aqueous solution was monitored using UV-Vis spectroscopy. Spectral measurements were performed regularly over an extended period, and the superimposability of the spectra recorded over more than 8 months, along with the lack of precipitation, confirmed the excellent colloidal stability of the synthesized nanoparticles, highlighting the effective stabilizing role of the PVP coating.

**Figure 3:**
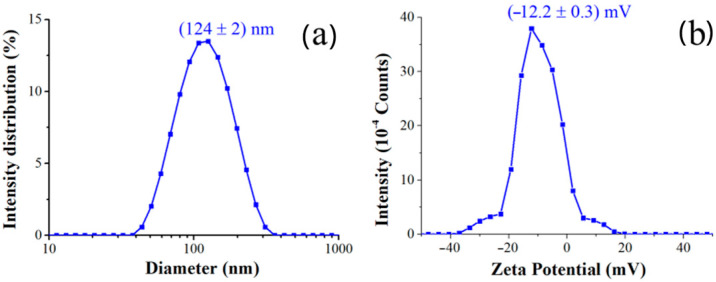
(**a**) DLS and (**b**) Zeta Potential measurements of AgNPs@PVP nanocomposites. Experimental conditions: Ag concentration of 0.02 mg/mL.

Dynamic Light Scattering (DLS) was utilized to determine the hydrodynamic size and size distribution of the synthesized nanocomposites. As summarized in Table 1 and in Figure 3, the AgNPs@PVP nanocomposites exhibited an average hydrodynamic diameter (Figure 3a) of (124 ± 2) nm and Zeta Potential of (−12.2 ± 0.3) mV (Figure 3b). The polydispersity index of 0.2 confirms a relatively narrow size distribution for the main population, indicating good homogeneity of the synthesized nanocomposites.

Transmission Electron Microscopy (TEM) was employed to evaluate the shape and dimensions of AgNPs, which were drop-cast onto a carbon-coated copper grid from properly diluted ethanol solutions.

**Figure 4:**
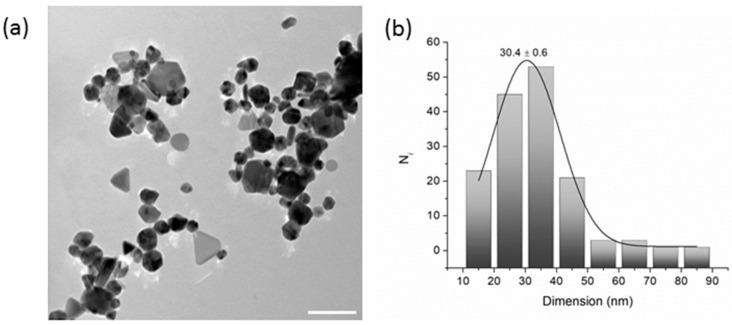
(**a**) TEM image (scale bar: 100 nm) and (**b**) size distribution histogram (*n* = 150) of AgNPs.

As shown in Figure 4a, the coexistence of spherical particle agglomerates with some triangular and polygonal silver nanostructures is evident. Commonly, this feature has been attributed to the Ostwald ripening process and to a contribution of PVP to the shape evolution process [36]. The size distribution analysis (*n* = 150) was fitted with a Gaussian function yielding a value of 30.4 ± 0.6 nm (Figure 4b).

### 2.2. Development and Characterization of Electrospun PVP and PVP+AgNPs Scaffolds by Electrospinning

To develop functional materials with antimicrobial properties, this study incorporated AgNPs into a PVP matrix (PVP+AgNPs) to enhance the antimicrobial performance of electrospun materials. Initial attempts using ethanol as the solvent for the electrospinning solution were unsuccessful due to its rapid evaporation, resulting in polymer buildup at the spinneret. Given PVP’s hydrophilicity, water was introduced as a co-solvent. However, its use had to be limited because increased surface tension negatively affected both the electrospinning process and fiber morphology [37]. The AgNPs remained stable in the selected solvent system, resulting in a consistent electrospinning process and scaffolds with a uniform macroscopic structure.

**Figure 5:**
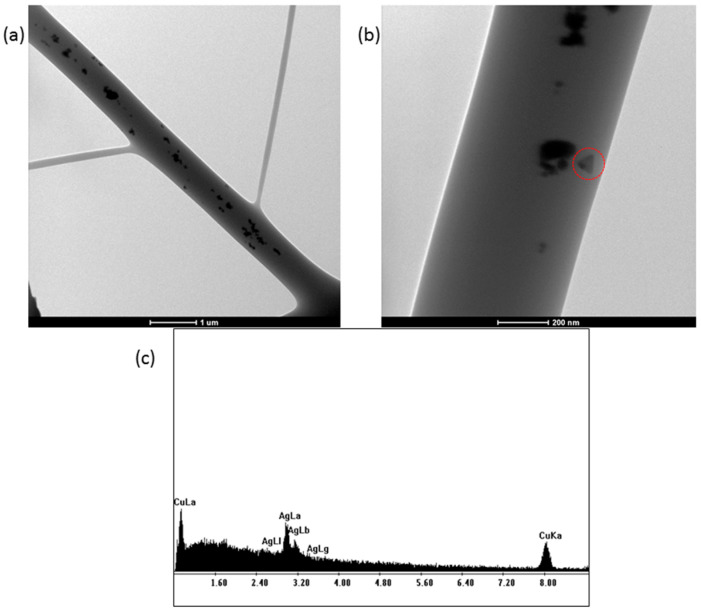
TEM images of PVP+AgNPs 0.20% *w*/*w* electrospun scaffold showing embedded AgNPs at different magnifications. The red circle highlights a triangular-shaped silver nanoparticle. Scale bars: (**a**) 1 µm; (**b**) 200 nm. (**c**) EDS spectrum showing characteristic peaks of silver (AgNPs) and copper (TEM grid).

TEM images of the electrospun PVP+AgNPs scaffolds were obtained by collecting fibers onto copper grids during the early stage of electrospinning. As shown in Figure 5, the silver nanostructures within the fibers appear evenly distributed, with clear evidence of triangular-shaped nanoparticles (Figure 5b), consistent with those previously observed in Figure 4a. Additionally, an EDS analysis of the PVP+AgNPs 0.20% nanofibers (Figure 5c) reveals a characteristic silver peak at approximately 3 keV, confirming the presence of AgNPs. The observed copper peak originates from the TEM grid.

**Figure 6:**
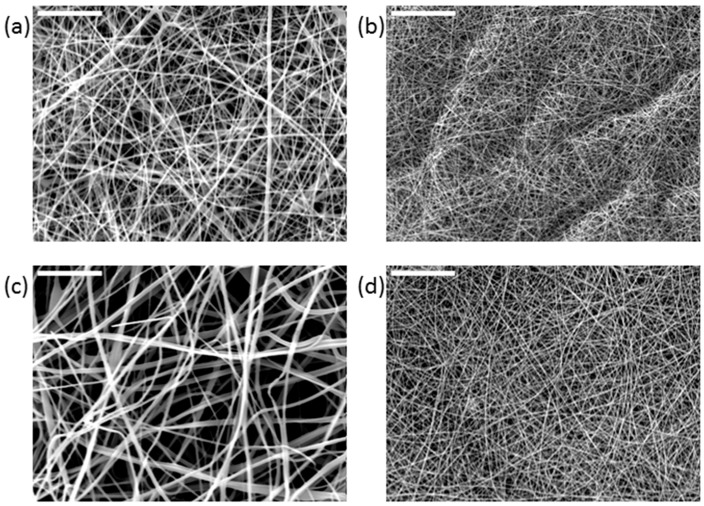
SEM images of (**a**) PVP; (**b**) PVP+AgNPs 0.05%; (**c**) PVP+AgNPs 0.10%; and (**d**) PVP+AgNPs 0.20% electrospun scaffolds showing morphology of the nanofibers. Scale bars: 50 μm.

The morphology of the scaffolds was investigated by scanning electron microscopy (SEM), with particular attention paid to the assessment of any defects (beads) and the fibers’ orientation and diameter distribution. The SEM images of the electrospun scaffolds (Figure 6) showed three-dimensional microstructures enriched with interconnected pores.

Studies indicate that the response of a cell relies on the elastic or viscoelastic resistance of a substrate, meaning that a cell’s adhesion and proliferation on a specific surface depend on the mechanical characteristics of the substrates at a nanometer scale. Thus, precise and extremely localized (at the nanometer level) assessments of mechanical surface attributes are essential to comprehend cellular reactions that may or may not replicate a specific tissue’s elasticity [38].

Thus, to examine the mechanical local characteristics of the fibrous electrospun scaffolds obtained in this study, force spectroscopy—a particular application mode of Atomic Force Microscopy (AFM)—was employed. AFM is, without a doubt, a potent and highly beneficial technique that has evolved since its beginning, incorporating various scanning modes and modules that enhance its functionalities by leveraging the diverse interactions between the tip and the sample surface.

The qualitative differences in local surface characteristics, including elastic modulus, have been investigated by measuring the force–distance curves, which relate the forces between the tip and sample to their separation distance. Specifically, the cantilever’s deflection is measured as the tip moves along the *z* axis. The sample’s reaction to an indentation force was used to specifically determine the Young’s modulus of electrospun scaffolds, which represents their surface stiffness. The literature indicates that this data is essential for comprehending cell behavior on the electrospun scaffolds.

**Figure 7:**
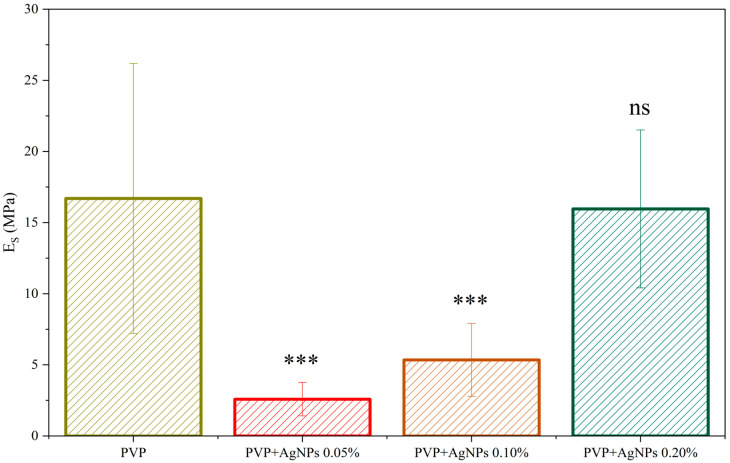
Elastic modulus (Es) of PVP and PVP-based scaffolds with varying concentrations of AgNPs. Bars represent mean values ± standard deviation. Statistical significance was assessed by F-test on variances (pF) followed, when appropriate, by Welch’s *t*-test (pt). Fibers with 0.05% and 0.10% AgNPs showed a highly significant reduction compared to neat PVP (*** *p* < 0.001), while fibers with 0.20% AgNPs were not significantly different from PVP (ns, *p* = 0.33). All AgNPs-containing samples were significantly different from each other (*** *p* < 0.001).

The stiffness was derived from the gathered force–distance curves by fitting the slope in the elastic region using the Derjaguin–Muller–Toporov (DMT) model. The value distribution of Young’s Modulus, Es, for all the investigated samples, is shown in Figure S1, displaying a broad but regular Gaussian-like distribution. For neat PVP electrospun fibers, a stiffness value of (16.7 ± 9.5) MPa has been recorded, with the relatively large standard deviation likely arising from the non-uniform fiber distribution. Interestingly, the incorporation of a very low concentration of AgNPs, at 0.05%, leads to a noticeable reduction in the average Young’s modulus to (2.6 ± 1.1) MPa. Even at such low concentrations, the presence of AgNPs appears to affect the fiber formation process, potentially influencing the polymer chain arrangement. With increasing AgNPs content, a gradual rise in stiffness is observed, reaching values comparable to those of pristine PVP fibers at around 0.20% concentration. The observed mechanical behavior suggests that the presence of AgNPs may influence the internal structure or packing of the polymer matrix, in terms of the single fiber structure, molecular arrangement of the amorphous chains, fiber orientation, and interaction between the fibers, with these effects becoming more evident at higher nanoparticle loadings (Figure 7).

To confirm the interaction between PVP and AgNPs, an ATR FT-IR analysis was conducted by comparing the spectra of neat PVP electrospun scaffolds and those containing 0.20% AgNPs (Figure S2). Both the pure PVP scaffold (black curve) and the PVP+AgNPs 0.20% scaffold (red curve) exhibited characteristic bands corresponding to amide I C=O stretching vibrations in the 1713–1569 cm^−1^ region. However, the amide I peak shifted from approximately 1644 cm^−1^ to 1653 cm^−1^ in the presence of AgNPs, indicating an interaction between PVP and the nanoparticles [39]. Additionally, a decrease in the intensity of the bands associated with amide III C–N stretching vibrations (1275–1288 cm^−1^) was observed, further supporting the presence of physical interactions between these functional groups and AgNPs, as also reported in the literature [40].

### 2.3. Biocompatibility Assessment and Cell Proliferation Analysis of Electrospun PVP and PVP+AgNPs Scaffolds Using L929 Fibroblasts Cells

**Figure 8:**
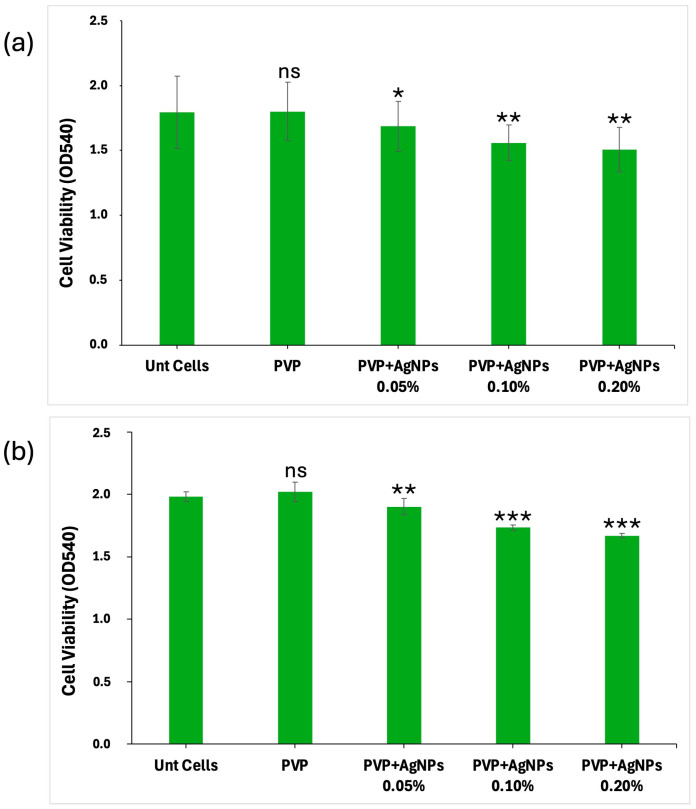
Cell viability of L929 cells cultured on pure PVP and PVP+AgNPs scaffolds at varying concentrations. Cell viability was assessed after (**a**) 24 h and (**b**) 48 h of incubation with pure PVP and PVP embedded with AgNPs at concentrations of 0.05%, 0.10%, and 0.20%. Bar graphs show the percentage of viable cells relative to the control group (pure PVP). Data are expressed as mean values of optical density (OD_540_) ± standard deviation. Statistical significance was performed using one-way ANOVA followed by Dunnett’s post hoc test. * *p* < 0.05, ** *p* < 0.01, and *** *p* < 0.001 (vs. pure PVP). ns = not significant.

The biocompatibility of the electrospun scaffolds was evaluated by analyzing their effect on L929 fibroblast cell viability and proliferation over 24 and 48 h. Both pure PVP and PVP-based scaffolds, incorporating AgNPs at varying concentrations (0.05%, 0.10%, and 0.20%), were assessed. All PVP scaffolds showed a good capability to interact with L929 cells, with their biocompatibility being related to AgNPs concentrations in a dose-dependent manner (Figure 8). Untreated cells (Unt Cells) and pure PVP scaffolds, used as controls, exhibited no cytotoxic effects, maintaining a high cell viability at both 24 and 48 h (~100% and 102%, respectively). For scaffolds containing 0.05% AgNPs, the cell viability was reduced to ~95% at 24 h and ~94% at 48 h, corresponding to a slight decrease relative to the controls. A more pronounced effect was observed with 0.10% AgNPs, where cell viability was reduced to ~86% at both time points, indicating a 14% decrease. The most significant reduction was seen with PVP+AgNPs 0.20% scaffolds, where cell viability decreased to ~84% at 24 h and ~82% at 48 h, corresponding to 16% and 18% reductions, respectively, compared to the controls. A statistical analysis indicated that the addition of AgNPs resulted in a statistically significant decrease in all treatment groups compared to the pure PVP scaffold. The effect was dose-dependent, with greater reductions observed at higher AgNPs concentrations, as confirmed by Dunnett’s test.

The cell viability data clearly indicate a concentration-dependent cytotoxic response to AgNPs exposure. While PVP scaffolds containing 0.05% AgNPs maintained relatively high levels of cell viability, increased concentrations of 0.10% and 0.20% led to statistically significant decreases, particularly after 48 h. These findings underline that, despite their beneficial antimicrobial effects, AgNPs exert considerable cellular stress at higher concentrations. The results are consistent with the previous literature highlighting AgNP-mediated oxidative stress, membrane damage, or nanoparticle-induced apoptosis as plausible mechanisms of cytotoxicity [41,42]. Nevertheless, as overall cell viability remained above 84% following 48 h of exposure, all scaffold formulations can be considered biocompatible under ISO 10993-5:2009 standards [43] for the biological evaluation of medical devices. According to these guidelines, materials are classified as non-cytotoxic if they sustain a cell viability above 70% in in vitro assays, affirming the suitability of the pure PVP and PVP+AgNPs electrospun scaffolds for biomedical applications.

**Figure 9:**
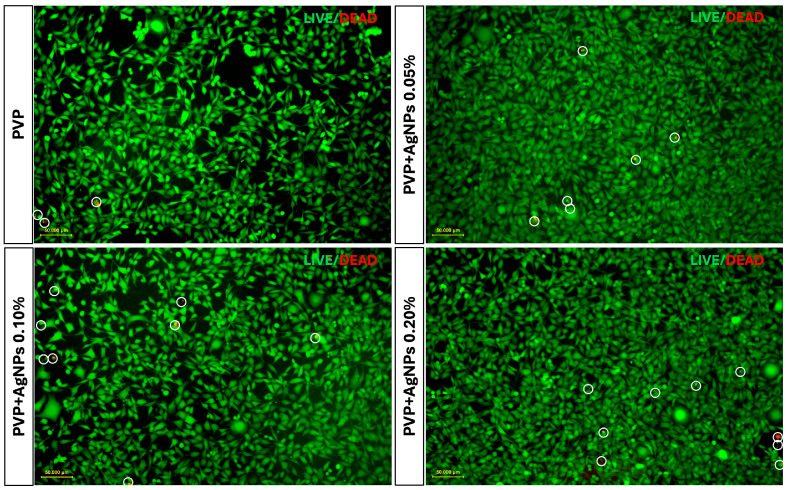
Live/Dead assay of L929 fibroblasts cultured on electrospun pure PVP and PVP+AgNPs scaffolds at 24 and 48 h. Representative fluorescence microscopy images show cells grown on pure PVP and PVP scaffolds containing AgNPs at 0.05%, 0.10%, and 0.20% concentrations. Green fluorescence indicated viable cells due to Calcein AM staining, while red fluorescence marked cell death due to BOBO-3 Iodide labeling. All fluorescence microscopy images include a scale bar of 50 µm.

To further assess the cellular response, Live/Dead fluorescence staining was performed on L929 fibroblasts cultured for 24 and 48 h on various PVP-based electrospun scaffolds. The imaging showed a progressive increase in red fluorescence intensity in PVP scaffolds containing 0.05%, 0.10%, and 0.20% AgNPs, indicating a concentration-dependent rise in cytotoxicity compared to pure PVP scaffolds (Figure 9).

**Figure 10:**
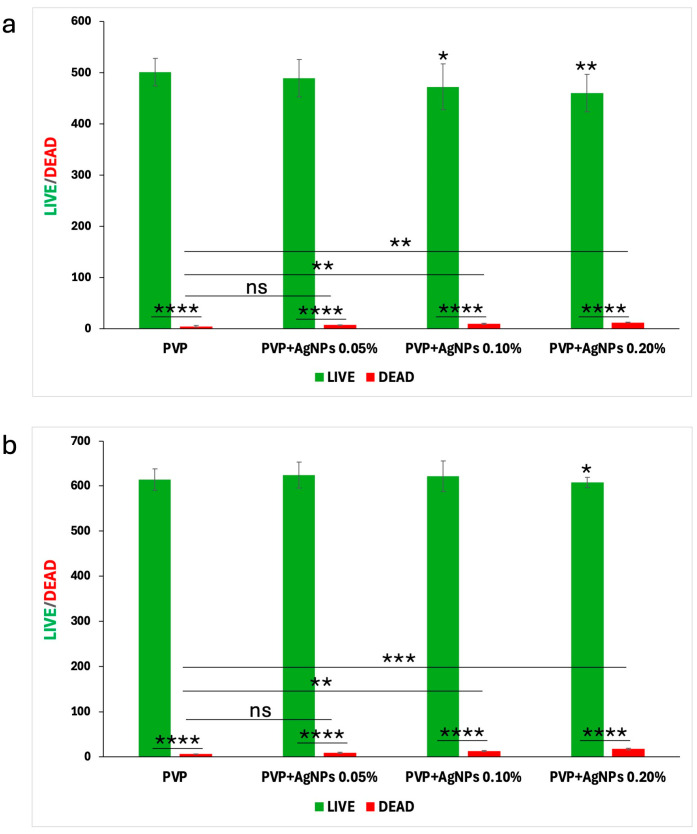
Quantitative analysis of Live/Dead L929 cells following culture on electrospun pure PVP and PVP+AgNPs scaffolds for 24 and 48 h. Bar graphs in panels (**a**) 24 h and (**b**) 48 h show the number of live (green) and dead (red) cells stained with Calcein AM and BOBO-3 Iodide, respectively. Statistical analysis was performed using two-way ANOVA to assess the effects of different AgNPs concentrations and cell status on cell count, followed by Tukey’s multiple comparisons test. Data are presented as mean ± SD, with statistical significance indicated as * *p* < 0.05, ** *p* < 0.01, *** *p* < 0.001, and **** *p* < 0.001. ns = not significant.

To quantify these fluorescence images, a Live/Dead cell count was performed, with the results at 24 and 48 h illustrated in the bar graphs shown in Figure 10, panels a and b, respectively.

Pure PVP scaffolds exhibited the highest number of live cells and minimal presence of dead cells, confirming their excellent biocompatibility. PVP scaffolds containing 0.05% AgNPs maintained a high rate of cell viability, with only a slight increase in cell death. In contrast, the incorporation of 0.10% and 0.20% AgNPs led to a moderate reduction in viable cells accompanied by a corresponding increase of approximately 1–2% in dead cells, indicating the concentration-dependent enhancement in cytotoxic effects relative to pure PVP.

However, the percentage of dead cells observed in all PVP+AgNPs scaffolds remained relatively low, ranging from 1.44% to 2.88% relative to the total viable cells, thereby reinforcing their overall biocompatibility despite the observed concentration-dependent cytotoxic response.

### 2.4. Evaluation of the Antibiofilm Activity of Electrospun PVP and PVP+AgNPs Scaffolds Against S. aureus and K. pneumoniae Planktonic and Sessile Cells

To assess the antibiofilm properties of the electrospun scaffolds, *S. aureus* ATCC 6538 and *K. pneumoniae* DSM 26371 were selected as the biofilm-forming strains [44,45]. The results demonstrated that scaffolds exhibited notable inhibitory effects against *S. aureus* and *K. pneumoniae* biofilm formation.

**Figure 11:**
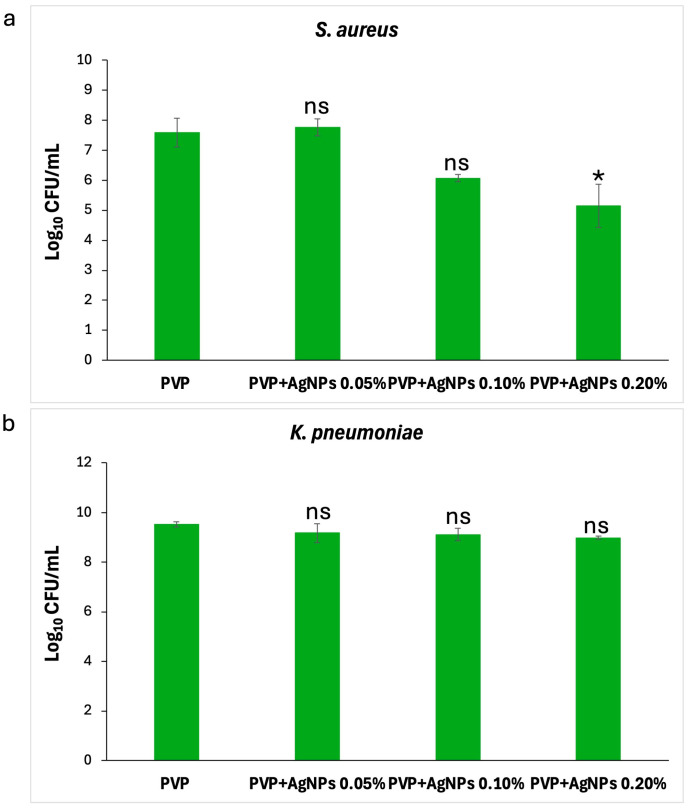
Impact of PVP+AgNPs scaffolds on planktonic growth of *S. aureus* and *K. pneumoniae.* Graphs show the bacterial response to pure PVP and PVP+AgNPs scaffolds at 0.05%, 0.10%, and 0.20% concentrations. (**a**) A concentration-dependent Log reduction in *S. aureus* growth was observed, with statistically significant differences correlating with increasing AgNPs concentrations (* *p* < 0.05). (**b**) No significant (ns) reduction was noted for *K. pneumoniae*. Statistical analysis was performed using the Kruskal–Wallis test, followed by Dunn’s post hoc test.

Effect on planktonic cells. As illustrated in Figure 11, the PVP+AgNPs 0.20% scaffold showed the strongest antibacterial activity against *S. aureus*, followed by PVP+AgNPs 0.10%. In contrast, PVP+AgNPs 0.05% and pure PVP scaffolds displayed similar and comparatively lower levels of activity. The numbers of planktonic *S. aureus* cells decreased from 7.8 to 6.1 log_10_ CFU/mL with pure PVP, PVP+s 0.05%, and PVP+AgNPs 0.10% scaffolds, while the PVP+AgNPs 0.20% scaffold reduced cell counts further to 5.5 log_10_ CFU/mL (Figure 11a). Statistical analysis revealed a significant difference only between pure PVP and PVP+AgNPs 0.20% scaffolds (*p* = 0.04), confirming a concentration-dependent antibacterial effect. In contrast, no statistically significant differences were observed in the planktonic growth of *K. pneumoniae* across all PVP+AgNPs scaffolds (Figure 11b). Cell counts were 9.52 log_10_ CFU/mL for pure PVP, and slightly reduced to 9.18, 9.12, and 8.99 log_10_ CFU/mL for PVP+AgNPs scaffolds at 0.05%, 0.10%, and 0.20%, respectively.

**Figure 12:**
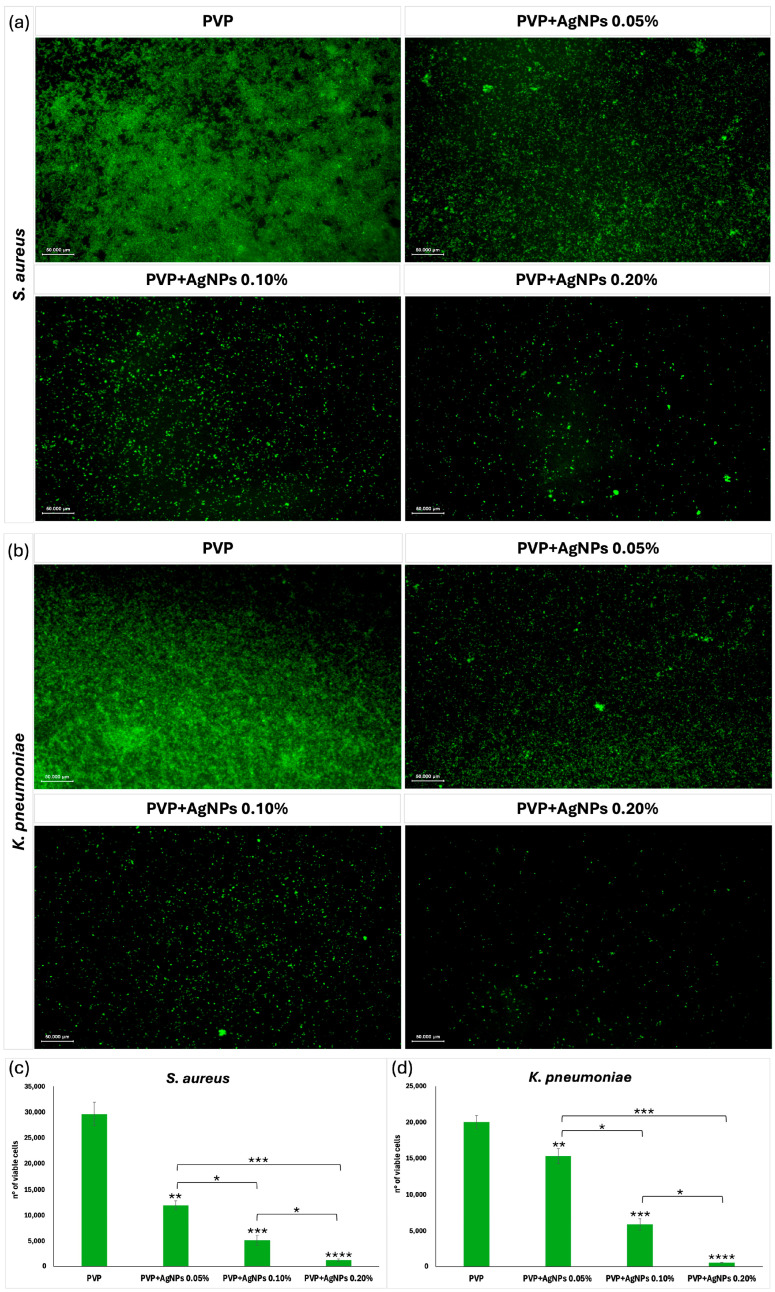
(**a**,**b**) Fluorescence microscopy images showing the viability of *Staphylococcus aureus* ATCC 6538 and *Klebsiella pneumoniae* DSM 26371 biofilms cultured on pure PVP and PVP embedded with AgNPs at concentrations of 0.05%, 0.10%, and 0.20%. Viable cells are indicated by green fluorescence, which decreases with increasing AgNPs concentrations. All fluorescence microscopy images include a scale bar of 50 µm. (**c**,**d**) Quantitative analysis of live cell counts for each group. Data are presented as mean ± SD. Statistical significance was determined using one-way ANOVA, followed by Tukey’s multiple comparisons test. * *p* < 0.05, ** *p* < 0.01, *** *p* < 0.001, and **** *p* < 0.0001 (vs. pure PVP).

Effect on sessile cells. To evaluate the antibacterial efficacy of electrospun scaffolds against sessile *S. aureus* and *K. pneumoniae* cells, Calcein AM fluorescence staining was carried out using scaffolds composed of pure PVP and PVP incorporating AgNPs at concentrations of 0.05%, 0.10%, and 0.20% (Figure 12). Given the hydrated and porous nature of PVP electrospun scaffolds, Calcein AM staining enabled the high-resolution visualization of live biofilm cells directly on the scaffold surface. This approach allowed for the precise assessment of antimicrobial efficacy, particularly in evaluating the bactericidal impact of AgNPs incorporation within the nanofiber matrix. Fluorescence microscopy (Figure 12a,b) revealed viable bacterial cells as green fluorescent signals, with the quantitative analysis shown in Figure 12c,d. Pure PVP scaffolds exhibited intense green fluorescence and the highest number of viable cells (~29,622 ± 2273 for *S. aureus*, ~20,039 ± 896 for *K. pneumoniae*), indicating minimal antimicrobial activity. The inclusion of AgNPs at 0.05% led to a noticeable decrease in fluorescence and viable cell counts (~12,005 ± 802 for *S. aureus*, ~15,356 ± 1022 for *K. pneumoniae*), suggesting mild antimicrobial activity. A further reduction was observed at 0.10% AgNPs (~5157 ± 975 for *S. aureus*, ~5932 ± 790 for *K. pneumoniae*), indicating moderate inhibition. The strongest antibacterial effect was demonstrated by the PVP+AgNPs 0.20% scaffolds, which exhibited minimal green fluorescence and the lowest viable cell count (~1244 ± 247 for *S. aureus*, ~556 ± 53 for *K. pneumoniae*). Statistical analysis revealed significant differences between PVP+AgNPs scaffolds (* *p* < 0.05, ** *p* < 0.01, *** *p* < 0.001, and **** *p* < 0.0001), confirming the concentration-dependent antimicrobial response.

These results together indicate that incorporating AgNPs into PVP scaffolds significantly reduces biofilm-forming *S. aureus* and *K. pneumoniae* in a concentration-dependent manner. Pure PVP, lacking inherent antimicrobial activity, does not inhibit bacterial growth; however, the gradual inclusion of AgNPs leads to a marked enhancement in antibacterial and antibiofilm performance. This improved efficacy aligns with the well-established mechanisms of AgNPs, which include the disruption of bacterial cell membrane integrity, the induction of oxidative stress through the generation of reactive oxygen species (ROS), and the interference with critical metabolic and replication pathways [46,47]. Moreover, AgNPs also change a biofilm’s architecture by distinct EPS-matrix formations [48].

Considering the persistent challenge of biofilm formation on medical device surfaces—a major contributor to healthcare-associated infections [49]—these multifaceted antimicrobial actions position PVP+AgNPs scaffolds as promising candidates for infection-resistant biomedical applications.

## 3. Materials and Methods

### 3.1. Materials

Silver acetylacetonate (Ag(acac)), polyvinylpyrrolidone (PVP), Sephadex G-25, absolute ethanol (EtOH), and ultrapure water (UPW) were purchased from VWR (Milan, Italy). 4-(2-hydroxyethyl)-1-piperazineethanesulfonic acid (HEPES) was purchased from Sigma-Aldrich (Merck Life Science S.r.l., Milan, Italy). All reagents were used without further purification. Centrifugal filters (Amicon^®^ Ultra, MWCO 3 kDa) were purchased from Sigma-Aldrich (St. Louis, MO, USA).

### 3.2. AgNPs@PVP Synthesis

A total of 0.5 g of PVP was weighed and dissolved in 100 mL of UPW. This mixture was stirred using a magnetic stirrer at 500 rpm for 15 min to ensure complete dissolution of the polymer. Subsequently, 4.1 mg of Ag(acac) was added to the solution, and the resulting mixture was continuously stirred magnetically for 24 h. A noticeable color change was observed within minutes after the addition of Ag(acac), with the solution transitioning to a pale yellow.

The purification of AgNPs from unreacted polymer and other salts was carried out by size exclusion chromatography (SEC) after drying the nanoparticles by rotary evaporation and redissolving them in 3 mL of a 10 mM HEPES solution (pH 6). A Sephadex G-25 column was packed and equilibrated with the same HEPES buffer. The concentrated AgNPs solution was then loaded onto the column and eluted with 10 mM HEPES solution, collecting only the colored fraction.

Following SEC purification, the HEPES buffer was removed from the nanoparticles by combining ultrafiltration (Amicon^®^ Ultra, MWCO 3 kDa; 5000 rpm, 30 min) (Merck Life Science S.r.l., Milan, Italy) and washing with UPW, a process repeated three times. AgNPs were then dried by rotary evaporation and resuspended in UPW (final Ag concentration 0.02 mg/mL) for carrying out the required characterizations or in EtOH (final Ag concentration: 0.75 mg/mL) for use in electrospinning experiments.

### 3.3. AgNPs Characterization

The optical properties of AgNPs colloidal solutions in UPW were recorded using a Stellarnet BLACK-Comet-SR diode-array spectrophotometer (StellarNet Inc., Tampa, FL, USA) equipped with fiber-coupled tungsten halogen lamps (Thorlabs mod. SLS201L, respectively) (Thorlabs Inc., Newton, NJ, USA), three multimodal fiber optic cables (Stellarnet F1000-UVVis-SR-1: length 1 m, Ø1000 µm core, multimode fiber, solarization resistant, and optical range: 190–1100 nm) (StellarNet, Inc., Tampa, FL, USA), a three-way fiber-coupled cuvette holder (Metrohm mod. DRP-CUV). The UV-Vis extinction spectra, recorded after purification in a 1 cm optical path length quartz cuvette (Hellma Italia, Milan, Italy), were corrected for water absorption. An in-line fiber-optic attenuator (Avantes mod. ATT-INL-EXT, Apeldoorn, Netherlands) was used to reduce the intensity of the transmitted light from the source within the sensitivity range of the detector.

Resonance Light Scattering (RLS) spectra were recorded using the above-described optical setup, with a right-angle geometry between incident and collected light [35]. RLS spectra were not corrected for the sample absorption.

The mean particle size and electrokinetic potential of AgNPs solutions were determined by the DLS and Zeta Potential techniques at 25 °C using folded capillary zeta cells and a Zetasizer Nano ZS instrument (Malvern Panalytical, Malvern, UK), equipped with a helium-neon 4 mW laser (λ = 632.8 nm) at a scattering angle of 173°. The values for the radium and electrokinetic potential of the AgNPs were obtained as a mean over three measurements, with the reported uncertainty being the average absolute deviation.

### 3.4. Electrospinning

The composition of the electrospinning solutions is reported in Table 1.

The preparation of the electrospinning solution/suspension was performed by the following general protocol: the AgNPs suspension in EtOH was diluted with additional EtOH solvent and stirred for 1 h at room temperature in the dark. Subsequently, the suspension was added to PVP, followed by the introduction of H_2_O as co-solvent. The resulting mixture was left stirring for 2 h at room temperature in the dark, then EtOH was added to reach the desired final volume, and the system was stirred overnight in the dark.

Electrospinning was performed on a Linari Engineering Gamma-High-Voltage generator electrospinning system. The polymer mixtures were loaded into a 10 mL glass syringe with an 18 G stainless steel needle and then electrospun at 19 kV with a regulated flow rate of 0.5 mL/h. The target was a round copper plate with a 90 mm diameter coated with aluminum foils, and the distance between the collector and the needle was fixed at 19 cm. To minimize temperature-related variations, all samples were electrospun in an air-conditioned room maintained at 22 °C. To reduce the influence of humidity, all samples were prepared within the same week under similar conditions (40–50% relative humidity).

### 3.5. Electrospun Fibers Characterization

TEM Analysis. AgNPs were diluted three-fold and deposited on carbon-coated copper grids (300 mesh). The grids were thoroughly rinsed with EtOH to remove the PVP coating agent and then air-dried before observation. The sample electrospun scaffold for TEM imaging was prepared by depositing fibers onto copper grids (400 mesh) for 5 min to ensure the deposition of only a few fibers onto the grid. TEM images were obtained using a Fei Tecnai G2 20 Twin transmission electron microscope (FEI Company, Hillsboro, OR, USA) at a 200 kV beam intensity.

SEM and EDS analysis. Fiber morphology of the electrospun scaffolds was observed by using a Philips-Fei ESEM XL30 scanning electron microscope (SEM) (FEI Company, Hillsboro, OR, USA), equipped with energy dispersive X-ray spectrometer (EDS). The samples were sputter-coated with gold before observation at the microscope operating at 20 kV. EDS analysis was conducted on carbon-sputtered samples.

AFM Analysis. The mechanical properties of the electrospun fibers were determined through force spectroscopy analyses employing AFM. Force–distance curves were obtained using the NTEGRA AFM (NT-MDT, Moscow, Russia), and the Young’s modulus was determined through the analysis of the elastic region of the acquired curves.

Stiff single-crystal silicon cantilevers with a symmetric tip shape were used (model Tap300AI-G, BudgetSensors, Sofia, Bulgaria: nom. frequency 300 kHz, nom. spring constant 40 N m^−1^, and tip radius < 10 nm). To calibrate the probe, the cantilever spring constant was measured by the Sader method [50]. The Young modulus was obtained by fitting experimental force–distance curves in the elastic region with Derjaguin–Muller–Toporov (DMT) [51] model, using the following Equation:

$$F+F_{ad}=\frac{4E_{s}}{3(1-\nu_{s}^{2})}R^{\frac{1}{2}}\delta^{\frac{3}{2}}$$

where *F* is the applied force; *F_ad_* is the adhesion force; *E_s_* is Young’s modulus; ν*_s_* is the Poisson’s ratio for the sample; *R* is the radius of the spherical indenter; and δ is the elastic indentation depth. A Poisson’s ratio of 0.40 was chosen based on values commonly reported for amorphous polymers such as PVP under standard conditions [52,53]. The measurements were made in triplicate, and more than three hundred curves were acquired for each sample by indenting each surface in different areas. The elastic modulus was obtained from the distribution of the collected data, assessed using Origin 2018 software.

ATR-FTIR Spectroscopy. ATR spectra were recorded on a model J- 460 instrument (Jasco Europe Srl, Cremella, Italy) equipped with an ATR PRO ONE Single-reflection ATR accessory using a single crystal diamond ATR prism. Spectra were acquired in the region from 4000 to 450 cm^−^^1^ with a spectral resolution of 2 cm^−^^1^ and 256 scans. Background spectra were recorded each time and then subtracted from the sample spectra.

### 3.6. Biocompatibility and Cell Proliferation Evaluation

To assess scaffold biocompatibility and cell growth, L929 murine fibroblasts were employed. L929 murine fibroblasts were obtained from the American Type Culture Collection (ATCC, Rockville, MD, USA). Cells were grown in Dulbecco’s Modified Eagle Medium High Glucose (DMEM, Merck Life Science S.r.l., Milan, Italy) with 10% fetal bovine serum (FBS, Merck Life Science S.r.l., Milan, Italy), 1% penicillin/streptomycin/amphotericin B (PSA, Merck Life Science S.r.l., Milan, Italy), maintained at 37 °C in a humid environment with 5% CO_2_, and subcultured until they reached 80% to 90% confluence. Electrospun scaffolds were made directly on 13 mm sterile glass coverslips. Following spinning, the scaffolds on coverslips were transferred to 24-well culture plate wells and UV-sterilized for 30 min on each side.

To guarantee initial adhesion, fibroblasts were carefully placed onto each scaffold at a concentration of 3 × 10^4^ cells per sample using a dropwise method. They were incubated for two hours under standard culture conditions. After that, 500 µL of fresh complete culture medium was gently added to each well to fully cover the samples without disturbing the cell placement. The scaffold compositions tested included PVP (10% *w*/*v*) and PVP mixed with AgNPs at 0.05%, 0.1%, and 0.2% (*w*/*w*).

Cell viability was measured by MTT [3-(4,5-dimethylthiazol-2-yl)-2,5-diphenyltetrazolium bromide] test (Merck Life Science S.r.l., Milan, Italy) at 24 and 48 h after seeding. For this, 500 µL of a 1 mg/mL MTT solution made in serum-free DMEM HG was added to each well and incubated for 2 h at 37 °C. The formazan crystals produced by living cells were then dissolved in 500 µL of Dimethyl Sulfoxide (DMSO, Merck Life Science S.r.l., Milan, Italy), and the absorbance was measured at 540 nm using a microplate reader (AMR-100 Biosigma, Verona, Italy). The viability data were normalized to cells seeded on PVP scaffolds, which served as the internal control.

To further evaluate scaffold cytocompatibility, Live/Dead staining was performed on L929 fibroblasts cultured on electrospun PVP and PVP+AgNPs scaffolds for 24 and 48 h. Following incubation, the culture medium was aspirated, and each scaffold was gently rinsed three times with phosphate-buffered saline (PBS, pH 7.4; Merck Life Science S.r.l., Milan, Italy). A 2× working solution of the LIVE/DEAD Cell Imaging Kit (Invitrogen, Cat. No. R37601; excitation/emission: 488/570 nm, Thermo Fisher Scientific, Waltham, MA, USA) was prepared according to the manufacturer’s instructions and applied to the samples. Scaffolds were then incubated with the staining solution for 30 min at room temperature in the dark. Fluorescence imaging was performed using a NEXCOPE NE 900 inverted fluorescence microscope (TiEsseLab S.r.l., Milan, Italy) to visualize viable cells (green fluorescence via Calcein AM) and dead cells (red fluorescence via BOBO-3 Iodide). Image acquisition was carried out using a digital camera, and quantification of live and dead cells was performed using Fiji ImageJ2 software (version 2.9.0/1.54f). For each scaffold type, a minimum of ten representative fields were analyzed to ensure robust statistical interpretation.

### 3.7. Activity on S. aureus and K. pneumoniae Biofilm Formation

The antibiofilm activity of electrospun scaffolds composed of pure PVP and PVP embedded with AgNPs at concentrations of 0.05%, 0.10%, and 0.20% was assessed. Scaffolds were fixed onto sterile glass coverslips and tested against *S. aureus* ATCC 6538 and *K. pneumoniae* DSM 26371. Bacterial strains were stored at −70 °C in Microbanks™ (Pro-lab Diagnostics, Neston, UK). To evaluate the antimicrobial efficacy, both planktonic and sessile (biofilm-associated) cells were analyzed following scaffold exposure.

Effect on planktonic cells. The scaffolds were sterilized under a UV lamp for 30 min and placed in sterile flat-bottom 24-well polystyrene microtiter plates (Corning Inc., Corning, NY, USA). Overnight cultures of *S. aureus* and *K. pneumoniae* in Tryptic Soy Broth (TSB; Merck Life Science S.r.l., Milan, Italy) supplemented with 1% glucose and TSB, respectively, were diluted to obtain an optical density of approximately 1 × 10^8^ CFU/mL. Then, 50 μL of this suspension was pipetted on each scaffold. All samples were incubated for 24 h at 37 °C in a humidified environment. Successively, each scaffold was washed three times in 200 μL of PBS to remove free-floating planktonic cells. The bacterial suspension obtained from the three washes was centrifuged at 12,000× *g* for 10 min and resuspended in 1000 μL of PBS. Serial ten-fold dilutions of the suspension were plated on Tryptic Soy Agar (TSA; Merck Life Science S.r.l.) plates and incubated for 24 h at 37 °C to detect colony-forming units (CFU/mL). Experiments were performed in triplicate and repeated three times.

Effect on sessile cells. Biofilms were cultivated on PVP electrospun scaffolds for 24 h at 37 °C under static conditions using *S. aureus* ATCC 6538 and *K. pneumoniae* DSM 26371 in TSB supplemented with 1% glucose and TSB, respectively. Following incubation, scaffolds were gently rinsed with phosphate-buffered saline (PBS) to remove planktonic cells. The viability of sessile bacterial cells adhered to pure PVP and PVP+AgNPs scaffolds was evaluated using Calcein AM fluorescence staining. Calcein AM was prepared at a final concentration of 2 µM in PBS and applied to the scaffolds for 30 min at 37 °C in the dark to allow intracellular esterase activation and conversion to fluorescent Calcein. After staining, scaffolds were rinsed again with PBS to remove excess dye and imaged using a NEXCOPE NE 900 inverted fluorescence microscope equipped with filter sets for green fluorescence (excitation/emission: 495/515 nm). Fluorescence intensity, indicative of viable biofilm cells, was quantified using Fiji ImageJ2 software (version 2.9.0/1.54f). For each scaffold condition, a minimum of ten randomly selected fields were analyzed to ensure statistical reliability and reproducibility.

### 3.8. Statistical Analysis

All data were evaluated either as raw values or expressed as mean ± standard deviation (SD), as appropriate. Differences among scaffold groups were assessed using one/two-way ANOVAs, followed by Dunnett’s or Tukey’s post hoc tests, and Kruskal–Wallis followed by Dunn post hoc tests for multiple comparisons. A *p*-value < 0.05 was considered statistically significant. Graphs were generated using GraphPad Prism software version 9.0 (GraphPad Software Inc., Boston, MA, USA) and Microsoft Excel. All experiments were performed in at least triplicate to ensure reproducibility and reliability.

## 4. Conclusions

This study demonstrates the successful fabrication and characterization of electrospun PVP scaffolds incorporated with AgNPs at varying concentrations. The results confirm that while pure PVP scaffolds exhibit excellent biocompatibility and antimicrobial activity, the incorporation of AgNPs imparts significant antibiofilm properties against *S. aureus* and *K. pneumoniae* strains. Live/Dead assays and quantitative analyses revealed a dose-dependent cytotoxic response in L929 fibroblasts, with higher concentrations of AgNPs exhibiting increased membrane damage and reduced cell viability. Nevertheless, all scaffold formulations maintained an overall cell viability above 85%, meeting the ISO 10993-5:2009 standard for cytocompatibility. These antimicrobial outcomes align with well-established mechanisms of AgNPs action, including membrane disruption, oxidative stress, and metabolic interference. Collectively, the data support the potential of PVP+AgNPs electrospun scaffolds as multifunctional biomaterials for wound healing and infection control. Further in vitro studies, particularly those involving a broader spectrum of pathogenic microorganisms, alongside long-term and in vivo investigations, will be crucial to comprehensively assess the interplay between antimicrobial efficacy and host tissue compatibility. These efforts will help define the therapeutic window, optimize scaffold performance, and ensure safe clinical translation.

## Figures and Tables

**Table 1:** Composition of the electrospinning solution.

Scaffold	PVP (% *w*/*v*)	AgNPs (% *w*/*w*)	H_2_O (mL)	EtOH (mL)	Final Volume (mL)
PVP	10.0	-	0.2	0.8	1.0
PVP+AgNPs 0.05%	10.7	0.05	0.2	0.8	1.0
PVP+AgNPs 0.10%	11.3	0.10	0.2	0.8	1.0
PVP+AgNPs 0.20%	12.7	0.20	0.2	0.8	1.0

## Data Availability

Data is contained within the article or Supplementary Materials.

## Supplementary Materials

The following supporting information can be downloaded at: https://www.mdpi.com/article/10.3390/ijms26189114/s1.

## Abbreviations

The following abbreviations are used in this manuscript:

AgNPs	Silver nanoparticles
PVP	Polyvinylpyrrolidone
TEM	Transmission electron microscopy
AFM	Atomic force microscopy
ECM	Extracellular matrix
*S. aureus*	*Staphylococcus aureus*
SPR	Surface plasmon resonance
UV-Vis	Ultraviolet-visible
DLS	Dynamic light scattering
UPW	Ultrapure water
HEPES	4-(2-hydroxyethyl)-1-piperazineethanesulfonic acid
SEC	Size exclusion chromatography
EtOH	Ethanol
DMEM HG	Dulbecco’s modified eagle medium high glucose
FBS	Foetal bovine serum
PSA	Penicillin/streptomycin/amphotericin B
MTT	3-(4,5-dimethylthia- zol-2-yl)-2,5-diphenyltetrazolium bromide
DMSO	Dimethyl sulfoxide
PBS	Phosphate-buffered saline
TSB	Tryptic soy broth
TSA	Tryptic soy agar
