# Supplementary material for: In Vitro Assessment of Electrospun PVP+AgNPs Scaffolds for Bioactive Medical Use
Source: Int J Mol Sci. 2025 Sep 18;26(18):9114. doi: 10.3390/ijms26189114 (PMC12470899; doi:10.3390/ijms26189114)
Supplement: Supplementary file 1 [file ijms-26-09114-s001.zip › ijms-3820280-supplementary.pdf]

Article

In Vitro Assessment of Electrospun PVP+AgNPs Scaffolds for Bioactive Medical Use

Ileana Ielo [1,†], Luana Vittoria Bauso [1,†], Antonio Laezza [2], Paola Campione [3], Luigi Fabiano [3], Martina Pastorello [1], Andreana Marino [1], Alessandro Laurita [2], Antonietta Pepe [2], Brigida Bochicchio [2], Giovanna De Luca [1,*], Grazia Maria Lucia Messina [3,‡] and Giovanna Calabrese [1,*,‡]

[1] Department of Chemical, Biological, Pharmaceutical and Environmental Sciences (ChiBioFarAm), University of Messina, Viale F. Stagno d'Alcontres 31, 98166 Messina, Italy; ileana.ielo1@unime.it (I.I.); luanavittoria.bauso@unime.it (L.V.B.); martina.pastorello@studenti.unime.it (M.P.); andreana.marino@unime.it (A.M.)

[2] Department of Basic and Applied Sciences (DISBA), University of Basilicata, Via Ateneo Lucano 10, 85100 Potenza, Italy; antonio.laezza@unibas.it (A.L.); alessandro.laurita@unibas.it (A.L.); antonietta.pepe@unibas.it (A.P.); brigida.bochicchio@unibas.it (B.B.)

[3] Department of Chemical Sciences, University of Catania and CSGI, Viale A. Doria 6, 95125 Catania, Italy; paola.campione@phd.unict.it (P.C.); luigi.fabiano@phd.unict.it (L.F.); gml.messina@unict.it (G.M.L.M.)

* Correspondence: giovanna.deluca@unime.it (G.D.L.); gcalabrese@unime.it (G.C.)

† These authors contributed equally to this work as co-first authors.

‡ These authors contributed equally to this work as co-last authors.

SUPPLEMENTARY MATERIALS

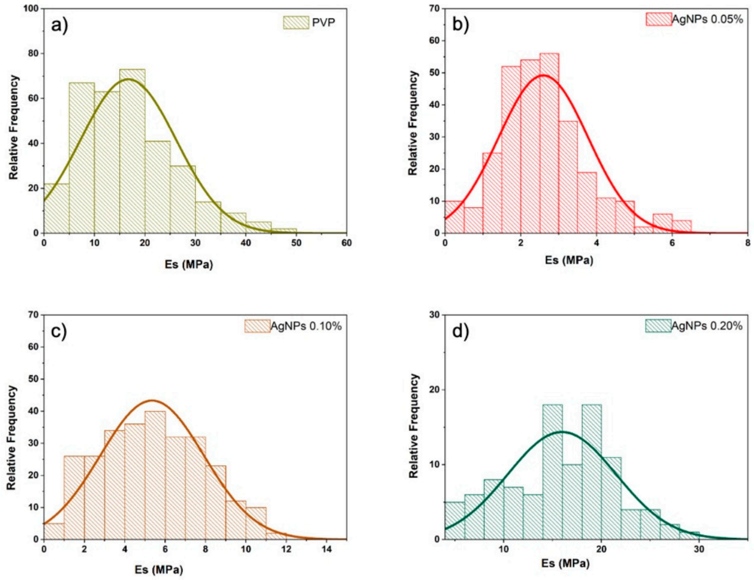

Figure S1. Young modulus distribution of: PVP (**a**); PVP+AgNPs 0.05% (**b**); PVP+AgNPs 0.10% (**c**); PVP+AgNPs 0.20% (**d**).

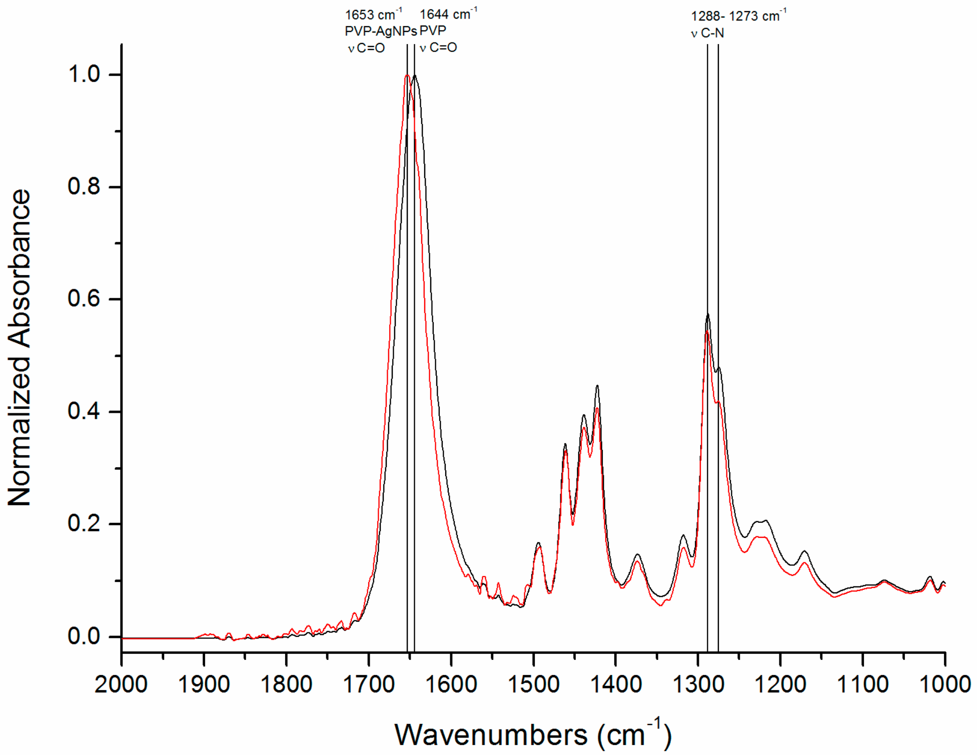

Figure S2. ATR FT-IR spectra of PVP (black curve) and PVP+AgNPs 0.20% (red curve).
